# Marked Direct Hyperbilirubinemia due to Ceftriaxone in an Adult with Sickle Cell Disease

**DOI:** 10.1155/2015/462165

**Published:** 2015-05-25

**Authors:** Daniyeh Khurram, Leonid Shamban, Robert Kornas, Maryann Paul

**Affiliations:** Department of Internal Medicine, Providence Hospital and Medical Center, 16001 W. Nile Mile Road, Southfield, MI 48075, USA

## Abstract

Drugs are a significant cause of liver injury. Drug-induced liver injury (DILI) can cause acute hepatitis, cholestasis, or a mixed pattern. Ceftriaxone is a commonly used antibiotic and has been associated with reversible biliary sludge, pseudolithiasis, and cholestasis. A 32-year-old male with sickle cell disease was admitted to the hospital for acute sickle cell crisis. On the second day of hospitalization, he developed cough and rhonchi with chest X-ray revealing right middle lobe infiltrates. Ceftriaxone and azithromycin were initiated. Subsequently, he developed conjugated hyperbilirubinemia and mild transaminitis. His total bilirubin trended upwards from 3.3 mg/dL on admission to 17 mg/dL. It was predominantly conjugated bilirubin, with preadmission bilirubin levels of 3-4 mg/dL. His transaminases were mildly elevated as well compared to previous levels. Extensive workup for bilirubin elevation was unremarkable. Ceftriaxone was switched to levofloxacin and the hyperbilirubinemia improved. On ambulatory follow-up, his bilirubin remained below 4 mg/dL. Ceftriaxone may be associated with marked direct hyperbilirubinemia particularly in sickle cell patients with chronic liver chemistry abnormalities. In the case of elevated bilirubin with concomitant ceftriaxone use, elimination of the offending agent should be considered.

## 1. Introduction

Medications account for approximately half of all cases of acute liver failure [1]. Drug-induced liver injury (DILI) can present with a wide range of acute and chronic liver diseases, including acute hepatitis, cholestasis, or a mixed pattern [2]. Many drugs have been associated with relatively high to very low rates of DILI; some of most common classes are acetaminophen products, statins, NSAIDs, and antibiotics [3]. Ceftriaxone, a third generation cephalosporin, is in widespread use due to its long half-life, broad-spectrum coverage, high tissue penetration rate, and favorable safety profile [4]. Ceftriaxone is known to cause cholestasis in neonates [5] and caution is urged for use in children due to reported cases of intrahepatic cholestasis [6]. Most of the documented cases of ceftriaxone associated hepatobiliary pathology are in neonates and children; few cases have been documented in adults [7]. Cases of ceftriaxone-induced liver injury in adults consist of reversible biliary sludge and pseudolithiasis [8]. We report a case of an adult male with an acute sickle cell crisis who developed significant conjugated hyperbilirubinemia, secondary to ceftriaxone administration.

## 2. Case

A 32-year-old obese African American male, with a history of sickle cell disease, presented to the hospital with severe pain in his legs and back that had become progressively worse for three days prior to presentation. For his sickle cell crisis, he has had multiple blood transfusions, but none within recent years. His medications consisted of hydroxyurea and morphine sulfate. His initial vital signs were heart rate of 72 beats per minute, blood pressure of 118/60 mmHg, respiratory rate of 18 breaths per minute, and temperature of 98.9 Fahrenheit. On examination, the patient was in distress and writhing in pain but otherwise no additional abnormalities were noted. According to the patient, these symptoms were typical of his sickle cell crisis.

Initial laboratory studies revealed a hemoglobin of 10 g/dL, reticulocyte count of 22%, haptoglobin of <10 mg/dL, and LDH of 462 units/L, and no electrolyte or kidney function abnormalities were noted. His total bilirubin on admission was 3.3 mg/dL (1.0 mg/dL upper limit of normal (UPLN)), AST was 51 unit/L (35 unit/L UPLN), ALT was 26 unit/L, ALP was 237 unit/L (129 unit/L UPLN), and GGT was 157 unit/L (60 unit/L UPLN) with albumin 4.1 gm/dL. His platelets and coagulation panel were within normal limits. Upon review of his prior records, it was determined that his liver chemistry panel was at baseline. Thus, based on the physical examination and laboratory findings, the patient was diagnosed with acute sickle cell crisis and was admitted to the hospital. He was started on a morphine (patient controlled analgesia) pump, hydroxyurea, intravenous fluids, and oxygen supplementation.

Initial chest X-ray done in the emergency room did not show any infiltrates or consolidations. However, on the second day, he developed cough and rhonchi in the right lung field on auscultation. Repeat chest X-ray revealed right middle lobe infiltrate and the patient was started on ceftriaxone and azithromycin for community acquired pneumonia.

**Figure 1:**
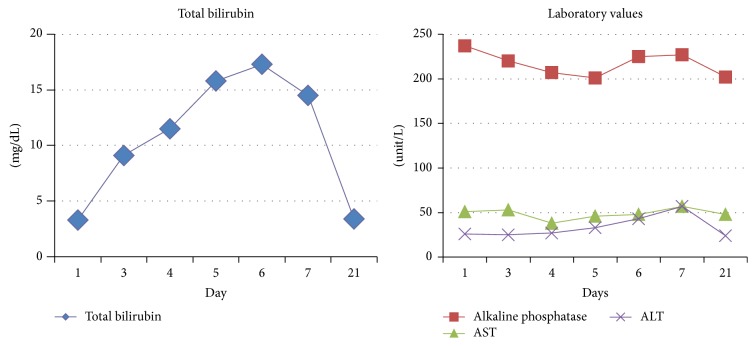
Trend of total bilirubin, alkaline phosphatase, AST, and ALT values from admission till outpatient follow-up.

Throughout the course of his hospitalization, the patient developed pronounced scleral icterus with acutely increased total bilirubin. There was minimal change in the other liver chemistries (Figure 1). A medication review was completed to investigate the cause of the direct hyperbilirubinemia. Ceftriaxone was continued since the most common adverse drug reaction with this medication reported is transaminitis. A complete additional workup was then pursued, as seen in Table 1. Ultrasonography of the liver and gallbladder did not demonstrate any abnormality. Furthermore, a subsequent ultrasound with additive arterial and venous evaluation did not reveal evidence of thrombosis.

After several days with intravenous hydration and appropriate pain control, the patient's pain symptoms returned to baseline. His laboratory values also improved, except for persistent hyperbilirubinemia that peaked at 17 mg/dL. His respiratory status improved as well. At this time, the ceftriaxone and azithromycin were changed to levofloxacin. Two days after changing the antibiotics, the total bilirubin started trending downwards to 15 mg/dL, and the patient was discharged with oral antibiotics. Repeat outpatient liver chemistries depicted total bilirubin levels returning to baseline values.

## 3. Discussion

Persistent liver chemistry elevations are consistent abnormalities seen in sickle cell patients [9]. Sickle cell disease has many effects on the hepatobiliary system [10] and can result in conjugated and unconjugated hyperbilirubinemia, with the most concerning condition being sickle cell intrahepatic cholestasis [11]. However, this is often a fatal condition associated with acute liver failure [12], requiring exchange transfusion.

Our patient developed marked elevation of his total and direct bilirubin in the hospital. This correlated with the administration of ceftriaxone. Extensive workup was done to exclude other etiologies. Review of his hematologist's records showed a chronic elevation of his total bilirubin with his highest documented reading at 4-5 mg/dL along with chronically mildly elevated ALT/AST levels in the range of 40–50 unit/L. He did not meet criteria for sickle cell intrahepatic cholestasis [11] because he did not have any significant elevation of his transaminases nor was there evidence of abnormal synthetic liver function. Furthermore, the rise in his bilirubin level did not correlate with an elevated LDH.

Ten percent of patients with sickle cell crisis can present with an acute sickle hepatic crisis [13]. These patients will have mildly elevated transaminase levels and a markedly increased serum bilirubin level [14]. These patients will also have significant right upper quadrant pain and generalized abdominal discomfort [11]. Our patient did not have these presenting symptoms; therefore, an alternative diagnosis was considered and pursued. The workup was negative, except for elevated ferritin and copper levels. A concern for secondary hemochromatosis was raised. Yet, the remainder of his iron studies and his blood transfusion history was not significant. The elevated ferritin and copper levels were attributed to an ongoing inflammatory process that has been described in numerous conditions [15]. Of note, there was no clinical evidence of Wilson's disease.

Although a definitive diagnosis of DILI due to ceftriaxone requires a tissue sample for analysis, a liver biopsy was not pursued. Percutaneous liver biopsy in sickle cell crisis has been associated with increased mortality, and studies recommend delaying the biopsy until the acute crisis has resolved if etiology is still in question [16]. Additionally, it is important to distinguish two similar conditions in sickle cell patients, benign hyperbilirubinemia and chronic intrahepatic cholestasis. Both these conditions appear with a significantly elevated direct bilirubin and mild elevated transaminases that can mimic ceftriaxone associated cholestasis [11]. Benign hyperbilirubinemia has been described in several pediatric patients on routine laboratory studies, while chronic intrahepatic cholestasis requires exchange transfusion for the bilirubin levels to decrease [11]. Our patient did not require exchange transfusion and his bilirubin began to improve within 48 hours of discontinuing ceftriaxone.

Of note, azithromycin has been associated with cholestasis-induced hepatitis [17]. Liver injury caused by azithromycin is typically cholestatic, arising within 1 to 3 weeks of starting treatment. It occasionally arises after azithromycin is stopped and can occur even after a short, 2- or 3-day course [18]. Recovery typically occurs within 4 to 8 weeks of stopping the medication [18].

The Naranjo Adverse Drug Reaction Probability Score was used to determine the probability of a drug interaction. Both azithromycin and ceftriaxone suggest the possibility of reaction [19]. However, his clinical course fits more with ceftriaxone. Ceftriaxone can cause biliary sludge and disease can arise within a few days of starting therapy [20]. Resolution of symptoms is typically rapid once ceftriaxone is stopped, although sludge and gallstones may be detectable by ultrasound for several months [20]. The time to resolution in our patient matches that of ceftriaxone, rather than azithromycin. Furthermore, the patient denied having been treated with ceftriaxone in the past. He did admit to using azithromycin for upper respiratory tract infection, supporting the possibility that ceftriaxone was the major drug involved.

## 4. Conclusion

This case highlights the possibility of ceftriaxone associated marked direct hyperbilirubinemia in young adults, especially in sickle cell patients with chronic liver chemistry abnormalities due to chronic hemolysis. In cases of elevated bilirubin in the presence of ceftriaxone, discontinuation of antibiotic should be considered and bilirubin levels should be trended until levels reach baseline. If levels do not return to baseline, other etiologies should be pursued. Furthermore, ceftriaxone should be used with caution in individuals predisposed to developing hyperbilirubinemia.

## Figures and Tables

**Table 1:** Serologic evaluation of elevated direct hyperbilirubinemia.

Serological evaluation of direct hyperbilirubinemia	Patient's value	Normal value
Serum copper level	243	72–166 mcg/dL
Ceruloplasmin	59	15–30 mg/dL
Ferritin	1,850	30–400 ng/mL
Iron	81	59–159 mcg/dL
Total Iron Binding Capacity (TIBC)	207	228–428 mcg/dL
Unsaturated Iron Binding Capacity (UIBC)	126	112–346 mcg/dL
Iron saturation	39	20–55%
Folate	9.6	3.1–17.5 ng/mL
Vitamin B12	697	211–946 pg/mL
Serum zinc	130	60–130 mcg/dL
Hepatitis A total	Negative	Negative
Hepatitis B core antibody IgM	Negative	Negative
Hepatitis B surface antibody	Negative	0.0–9.9 Iunits/mL
Hepatitis B surface antigen	Negative	Negative
Hepatitis C antibody	Negative	Negative
Total IgG	1480	700–1600 mg/dL
Total IgA	376	70–400 mg/dL
Total IgM	19	40–230 mg/dL
Antinuclear Antibody (ANA)	Negative	Negative
Mitochondrial antibody	Nondetected	Nondetected
Smooth muscle antibody	Nondetected	Nondetected
